# Evaluating the use of real-time data collection using SMS texts in the SIMS study

**DOI:** 10.1186/1745-6215-16-S2-O65

**Published:** 2015-11-16

**Authors:** Tracey Davidson, Alison McDonald, Gladys McPherson, John Norrie

**Affiliations:** 1 University of Aberdeen, Aberdeen, UK

## Background

A recent innovation in Patient Reported Outcomes (PROs) collection is the use of SMS texts.^1^ Results from a large randomised controlled trial (RCT) found this real-time data collection was both feasible and acceptable.

As part of the multicentre NIHR HTA funded SIMS study (Adjustable Anchored Single-Incision Mini-Slings Versus Standard Tension-Free Mid-Urethral Slings in the Surgical Management Of Female Stress Urinary Incontinence; A Pragmatic Multicentre Non-Inferiority Randomised Controlled Trial), we evaluated responses to a post-surgery pain diary comparing PROs collected via texts and paper. The study raised several interesting data collection.

## Method

Participants (n= 189) were provided with a pain diary to complete on the 14-days post-surgery. If participants consented to receive texts, they also received daily texts to report their pain score and any painkillers taken. Responses to texts were free of charge.

## Results

Results will be presented reporting response rates and comparison between pain scores between participants responding in both modes. The number of text responses that could not be matched to a text question will also be reported.

## Discussion

Texts were an acceptable mode of response to participants with over 66% (n=126) responding by text. The number of responses reported in both modes that were identical and a possible explanation of the discrepancy will be discussed.

Data collection challenges will also be discussed including: where responses cannot be matched to a text question what happens with the unmatched data? If there is a discrepancy between participant's responses in both modes which data do you use?

